# Supplementary material for: Integrating Single-Cell RNA-Seq and ATAC-Seq Analysis Reveals Uterine Cell Heterogeneity and Regulatory Networks Linked to Pimpled Eggs in Chickens
Source: Int J Mol Sci. 2024 Dec 15;25(24):13431. doi: 10.3390/ijms252413431 (PMC11679886; doi:10.3390/ijms252413431)
Supplement: Supplementary file 1 [file ijms-25-13431-s001.zip › Supplementary Figures S1-S3.pdf]

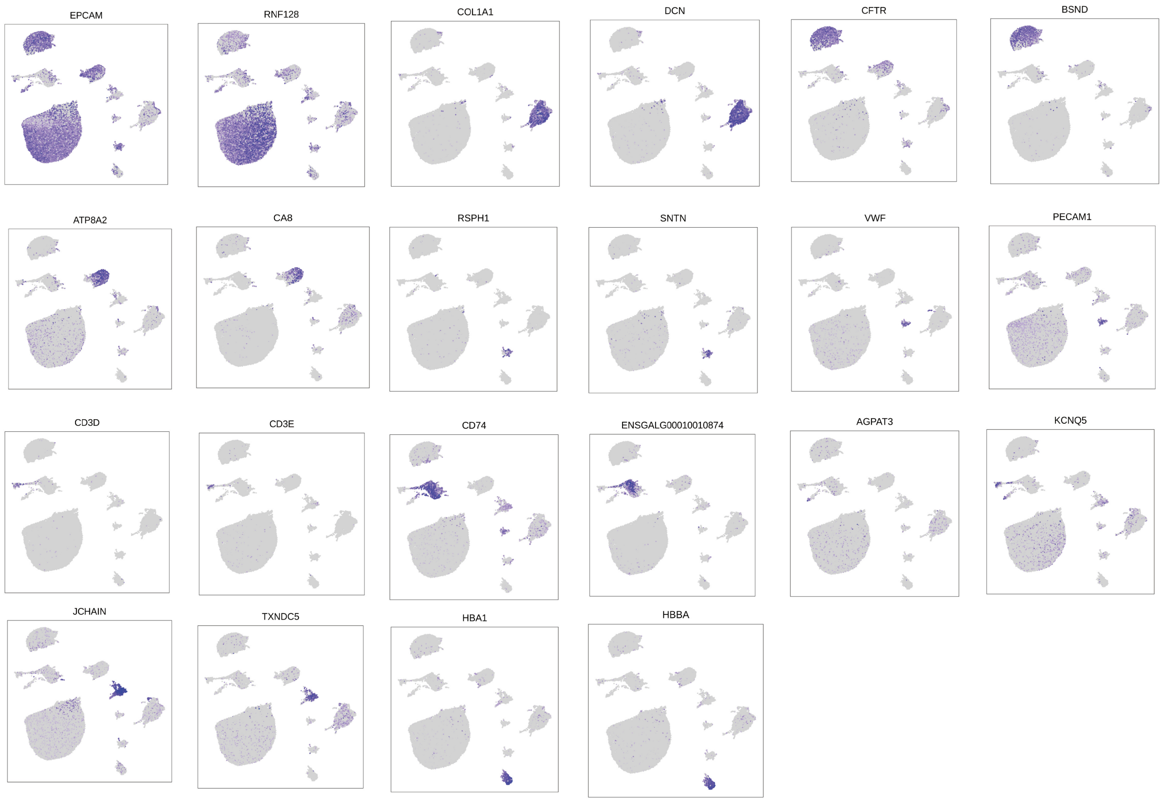

Figure S1 UMAP plot illustrating the expression patterns of marker genes used for cell type annotation. The cell types and their markers are as follows: epithelial cells (EPCAM, RNF128), fibroblasts (COL1A1, DCN), ionocytes (CFTR, BSND), luminal epithelial cells (ATP8A2, CA8), ciliated epithelial cells (RSPH1, SNTN), endothelial cells (VWF, PECAM1), T cells (CD3D, CD3E), macrophages (CD74, ENSGALG00010010874), natural killer cells (AGPAT3, KCNQ5), B cells (JCHAIN, TXNDC5), and erythrocytes (HBA1, HBBA).

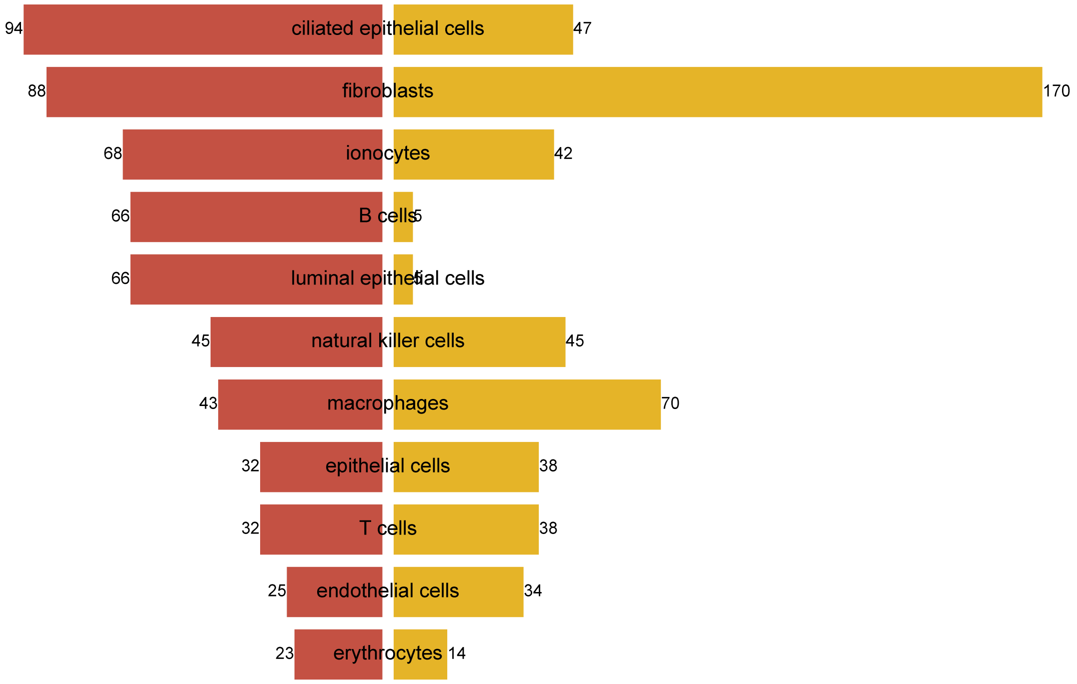

Figure S2 Bar chart showing the number of differentially expressed genes between cell types in NE and PE groups.

The left side represents the number of genes upregulated in NE group cells, while the right side represents the number of genes upregulated in PE group cells.

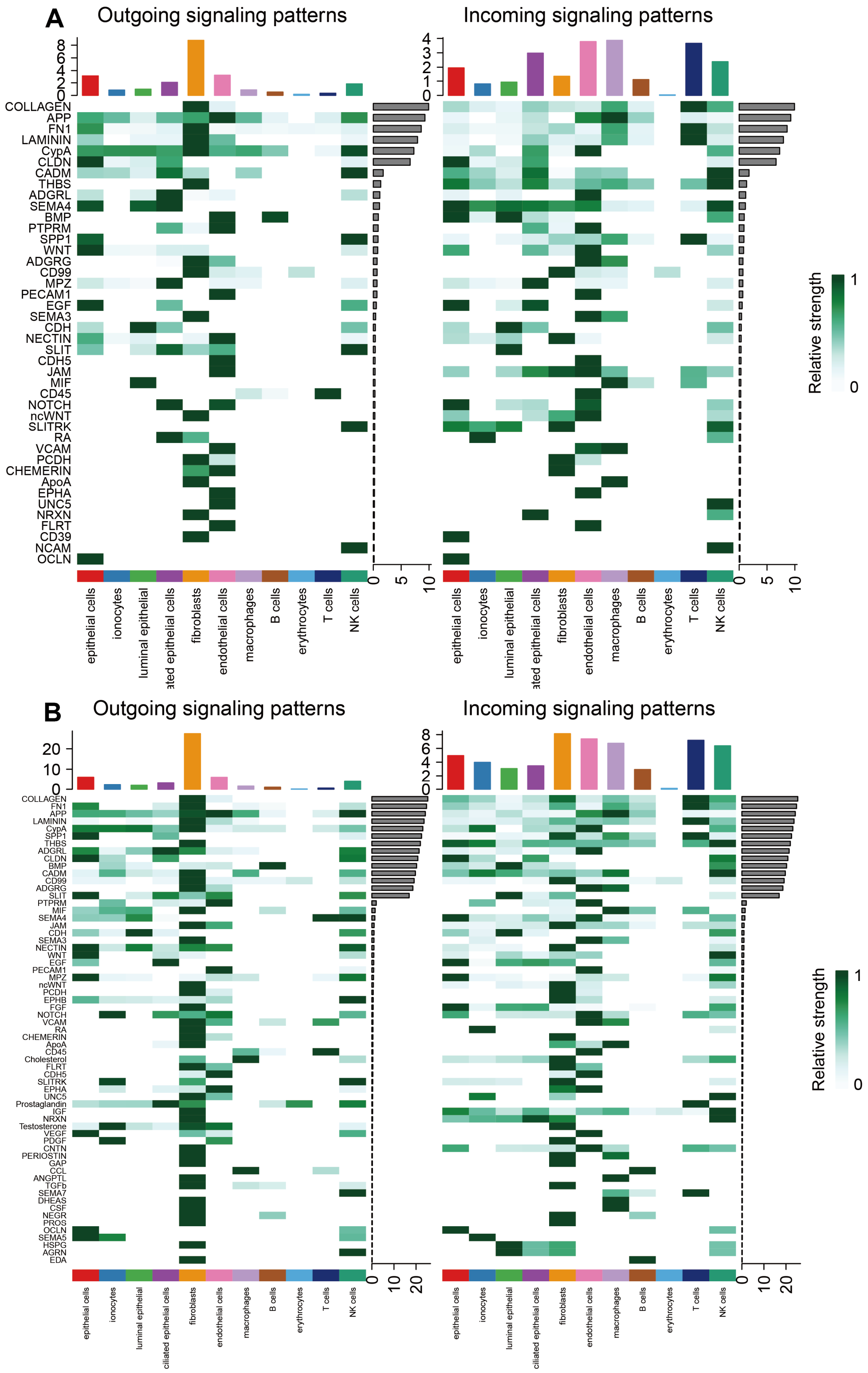

Figure S3 Visualization of the interaction strength between cells, as well as signaling patterns, in the NE and PE groups using a heatmap. In the heatmap of cell-cell interactions for the NE group (**A**) and the PE group (**B**), the x-axis represents cell types, while the y-axis denotes signaling pathways of interactions. The bar graph at the top indicates the cumulative interaction strength of all signaling pathways for each cell type, and the bar graph on the right represents the cumulative interaction strength of each signaling pathway. The left side of the heatmap

shows the outgoing signaling patterns, while the right side displays the incoming signaling patterns. The intensity of the color reflects the relative strength of the interactions, with darker colors indicating greater strength.
